# Total hip arthroplasty in patient with hip fracture and congenital pubic diastasis

**DOI:** 10.1308/rcsann.2025.0037

**Published:** 2025-06-17

**Authors:** K Saravanan, K Malik-Tabassum, A Rajpura, AK Gambhir

**Affiliations:** ^1^ The Queen’s College, University of Oxford, UK; ^2^ East Sussex Healthcare NHS Trust, UK; ^3^ Wrightington, Wigan and Leigh NHS Foundation Trust, UK

**Keywords:** Total hip arthroplasty, Fractures, Pelvic diastasis, Case report, Pubic diastasis

## Abstract

Congenital pelvic deformities, such as pubic diastasis, significantly complicate the surgical planning and execution of total hip arthroplasty (THA), the gold standard for treating degenerative hip disease unresponsive to conservative measures. These challenges are exacerbated in the context of trauma, because of disrupted anatomical landmarks and soft tissue imbalances. THA becomes particularly demanding in cases involving traumatic femoral neck fractures in patients with congenital anomalies of the pelvis. We present a unique case of a 62-year-old man with an acute, post-traumatic left-sided hip fracture, and a history of congenital bladder exstrophy and pubic diastasis, who successfully underwent THA. Meticulous preoperative planning with three-dimensional reconstruction software was essential in overcoming the anatomical challenges posed by this case, enabling our successful outcome.

## Background

Bladder exstrophy is a rare and complex congenital anomaly that affects both the urogenital and musculoskeletal systems.^1^ Characterised by incomplete closure of the lower abdominal wall during embryogenesis, this condition has a prevalence of approximately 1 in 30,000 live births.^2^ Musculoskeletal abnormalities associated with bladder exstrophy frequently include symphysis diastasis, hip dysplasia and rotational deformities, such as external rotation of the posterior pelvis and iliac wings, alongside acetabular retroversion. Shortening of the pubic bones is also a common feature, further complicating the anatomical landscape.

In managing bladder exstrophy, posterior or anterior bilateral pelvic osteotomies are often performed to facilitate bladder reconstruction, aiming to reduce tension on the anterior abdominal wall and prevent wound dehiscence. However, the decision to perform primary exstrophy closure with or without concurrent pelvic osteotomy typically depends on the surgeon’s preference rather than specific anatomical criteria. There is a paucity of data regarding outcomes in patients who have undergone bladder exstrophy repair without closure of the pelvic diastasis. In such cases, challenges such as acetabular retroversion, rotational deformities of the pelvis and pelvic ring instability pose significant hurdles for orthopaedic surgeons, particularly when these patients require surgical interventions like total hip arthroplasty (THA). Thorough preoperative planning in these rarer cases is therefore crucial in navigating around the anatomical challenges that underlie implant positioning in these patients and ensuring successful postoperative outcome.

THA remains the gold standard surgical treatment for most degenerative hip conditions.^3^ However, the presence of deformities such as femoral, acetabular or pubic anomalies, specifically pubic diastasis, significantly complicates the procedure. To our knowledge, only five cases in the literature have addressed the use of THA in patients with chronic pubic diastasis, secondary to either long-term post-traumatic injury or congenital bladder exstrophy.^4–8^ This case report presents a rare instance of an adult patient with a history of congenital bladder exstrophy who sustained an acute, post-traumatic left-sided hip fracture and subsequently underwent THA. To the best of our knowledge, this is the first documented case of THA performed on a patient with a post-traumatic hip fracture, pubic diastasis and a history of congenital bladder exstrophy.

## Case history

A 62-year-old man was referred to our tertiary referral centre following a displaced left-sided hip fracture sustained following a fall off a ladder. Initially admitted to a local district general hospital, the patient’s medical history included congenital bladder exstrophy, which was managed with a urostomy in the right iliac fossa. Despite this condition, he remained fully functional and continued his occupation as a builder.

**Figure 1:**
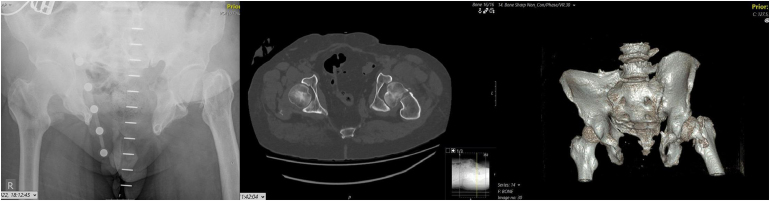
(a) Anteroposterior x-ray showing left-sided hip fracture. (b) Axial sequence of computed tomography (CT) pelvis imaging showing pubic diastasis and left-sided neck of the femur fracture. (c) Three-dimensional CT reconstruction of the pelvis

Upon presentation at our centre, the patient reported severe pain and immobility. X-rays revealed a pelvic diastasis alongside a left intracapsular neck of femur fracture (Figure 1a). Because of the abnormal anatomy, a computed tomography (CT) scan (Figure 1b) with three-dimensional (3D) reconstructions (Figure 1c) was performed to better understand the anatomical challenges. Given the case complexity, a multidisciplinary team discussion was held among the hip surgeons to discuss the optimal surgical strategy including planning, implant choice and fixation strategy. Based on previously published case reports involving chronic pelvic diastasis, uncemented cups were consistently used and not associated with adverse outcomes.^4–8^ Because chronic diastasis often results in a stable, remodelled pelvic configuration, an uncemented cup was deemed suitable. An uncemented Trident acetabular cup (Stryker) was chosen to allow for the use of a dual mobility articulation (MDM) which we felt offered additional benefit in terms of stability given the altered pelvic morphology.

**Figure 2:**
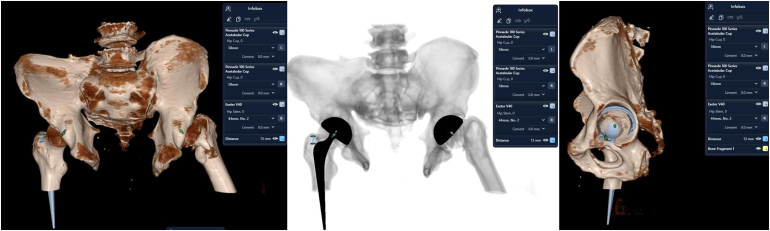
Surgical planning done using three-dimensional planning software for total hip replacement

Surgical planning involved the use of 3D planning software (Figure 2). The 3D planning enabled accurate templating of cup size, depth and orientation, while accounting for the altered bony landmarks due to chronic pelvic diastasis. Key anatomical landmarks used during planning included the anterior and posterior acetabular walls (for assessing version), the superior wall (for inclination) and the acetabular fossa (to guide depth and direction of reaming). The software also allowed us to anticipate how much of the anterior wall would remain visible following reaming, serving as an intraoperative confirmation of appropriate cup positioning. Because of the chronic pelvic diastasis and resultant external rotation of the pelvis, standard definitions of acetabular and femoral version – and thus combined version – were not directly applicable. Rather than targeting traditional values, we aimed to replicate this patient’s native bony anatomy. Preoperative axial views from the CT-based 3D planning showed that the native acetabulum had near-zero anteversion. We proceeded with the following planned intervention: a hybrid total hip replacement, incorporating a polished tapered cemented stem (Exeter stem) and an uncemented press-fit acetabular cup (Trident), with dual mobility construct.

In the operating theatre, the patient received spinal and general anaesthesia, with tranexamic acid and cefuroxime administered perioperatively. The patient was positioned laterally with a 15-degree anterior tilt to facilitate optimal visualisation of the posterior structures. A posterior approach was utilised. The superficial dissection commenced with an incision of the fascia lata to expose the vastus lateralis distally and the gluteus maximus fibres were split proximally. For deep dissection, the hip was internally rotated to stretch the short external rotators. The sciatic nerve was visualised and protected. Stay sutures were placed in the piriformis and obturator internus tendons, which were then detached close to their femoral insertions and reflected backwards to protect the sciatic nerve. The capsule was incised longitudinally, and the hip was dislocated with internal rotation following capsulotomy.

After extracting the fractured femoral head, the acetabulum was prepared and reamed according to the preoperative plan. The transverse acetabular ligament was identified, and both anterior and posterior walls were exposed to ensure precise reaming and cup placement. A press-fit acetabular cup (56mm Trident) was inserted with gentle impaction, followed by the insertion of a dual mobility liner (internal diameter 46mm). The femur was then prepared; the femoral anatomy on the contralateral side appeared normal on CT scan, and no specific modifications were required. A trial reduction was performed using a trial broach at 15-degree anterversion and a dual mobility trial head to assess length, offset and stability. Subsequently, a cement restrictor was placed in the femoral canal, followed by thorough washout and drying. The canal was cemented using a third-generation cementing technique, and the polished tapered stem (Exeter stem 44 no. 4) was inserted in the planned anteversion and depth. Once the cement had set, a second trial reduction was conducted before final implantation of definitive 28+4 ceramic with 28/46 dual mobility head. Posterior capsule and short external rotators were repaired with trans-osseous sutures. The wound was closed in layers using absorbable sutures for the skin.

**Figure 3:**
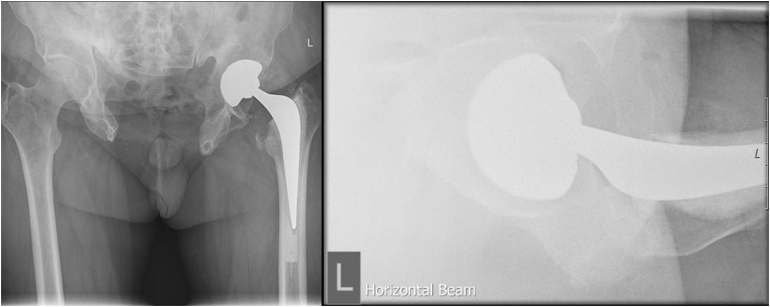
Postoperative anteroposterior and lateral x-rays of left hip total replacement

Postoperatively, the patient was managed according to the standard protocol; he received appropriate analgesia, was allowed full weight-bearing and was administered low molecular weight heparin. Physiotherapy commenced immediately, and the patient was discharged three days postoperatively. Follow-up assessments at 6 weeks, 3 months and 12 months demonstrated excellent recovery. At the 12-month follow-up, the patient reported no pain, exhibited a normal range of movement, walked unaided and had resumed his normal activities. X-rays confirmed good osseointegration of the acetabular cup, excellent cementation of the stem, and proper alignment and offset (Figure 3). The patient achieved an Oxford Hip Score of 39 of 48, indicating a successful outcome.^9^

## Discussion

This case highlights the complexities associated with performing THA in patients with congenital anomalies such as bladder exstrophy and pubic diastasis. The variability in pelvic anatomy, particularly in assessing and planning the acetabular cup version, presents significant challenges. However, the use of 3D planning software played a crucial role in preparing the case and visualising these anatomical complexities. The use of hybrid hip replacement with a dual mobility construct proved to be an excellent solution. The successful outcome in this case underscores the importance of meticulous preoperative planning and the strategic use of modern surgical techniques in managing complex THA cases.

## Ethical review statement

This study adhered to the ethical principles outlined by the UK Medical Research Council. This study was approved by the institutional review board at each participating hospital. Written informed consent was obtained from the patient for the publication of this case report and any accompanying images.

## Data availability statement

Data sharing is not applicable to this article as no datasets were generated or analysed during the current study.
